# Novel Four-gene Panel for Detecting Senescence-associated Cell States in Skeletal Muscle Tissue

**Published:** 2026-07-30

**Authors:** TARO KUNITOMI, YURI YAMASHITA, ERI ARIKAWA-HIRASAWA

**Affiliations:** 1 Research Institute for Diseases of Old Age, Juntendo University Graduate School of Medicine, Tokyo, Japan; 1 Research Institute for Diseases of Old Age, Juntendo University Graduate School of Medicine, Tokyo, Japan; 2 Department of Neurology, Faculty of Medicine, Juntendo University, Tokyo, Japan; 2 Department of Neurology, Faculty of Medicine, Juntendo University, Tokyo, Japan

**Keywords:** skeletal muscle, sarcopenia, cell senescence

## Abstract

**Objectives:**

Sarcopenia, characterized by age-associated loss of skeletal muscle mass, function, and physical performance, is a major challenge in aging societies because of its association with a decreased lifespan. Existing gene expression-based diagnostic methods often rely on large gene sets, requiring high costs and analytical complexity. In this study, we developed a machine learning-driven framework to identify senescence-associated cell states in skeletal muscle tissue.

**Methods:**

Publicly available single-cell RNA sequencing data from 2- and 24-month-old C57BL/6J male mice from single-cell and single-nucleus RNA sequencing datasets comprising over 365,000 cells from skeletal muscle were obtained from the DRYAD Repository and consolidated into 15 cell populations. Candidate genes for machine learning were selected from differential expression analysis. Multiple machine learning algorithms, including logistic regression, support vector machines, and random forest, were trained with recursive feature elimination. Model performance was evaluated using the area under the receiver operating characteristic curve.

**Results:**

Differential expression analysis across 15 distinct cell populations yielded 30 candidate genes, to which five machine learning models were applied to select biomarkers. Using our approach, we identified a four-gene panel (*Malat1*, *Wdr89*, *Zfp36*, and *Jund*) exhibiting high predictive accuracy. This panel was validated using additional aging datasets and compared with existing models, which highlighted its potential as a reliable tool for detecting senescence-associated cells.

**Conclusions:**

In this study, we established a four-gene biomarker panel for detecting senescence-associated cells in skeletal muscle, providing a practical tool for investigating sarcopenia pathophysiology and identifying therapeutic targets.

## Introduction

Sarcopenia, characterized by the loss of skeletal muscle mass, function, and physical performance with age, represents a pressing challenge in aging societies worldwide^1)^. It is associated with frailty, reduced mobility, and a higher risk of falls. Sarcopenia contributes to reduced quality of life among the elderly as well as rising healthcare burden in older populations^2)^. The number of individuals with sarcopenia in Europe has been predicted to increase to 72.4% by 2045, highlighting it as a growing public health challenge^3)^.

The identification of senescent cells within tissues is central to understanding and treating sarcopenia and other age-related diseases. Traditional approaches for detecting senescent cells rely on classical single markers, such as senescence-associated β- galactosidase (SA-β-Gal), which reflects increased lysosomal activity, and p16^INK4a^, a key cell cycle regulator that accumulates in response to stress signals^4, 5)^. These markers are widely used in histochemical and immunohistochemical assays. However, they have well-known limitations: SA-β-Gal can produce false positives in non-senescent cells such as macrophages, and p16^INK4a^ is often difficult to detect reliably in mouse tissue sections with the currently available antibodies^6)^.

Multi-marker approaches have been developed to overcome the limitations of single-marker evaluation. For example, the SenMayo signature, comprising 125 genes, enables broad detection of senescent cells across multiple tissues^7)^. Similarly, dozens to thousands of genes have been used to develop gene expression-based classifiers for senescence^8, 9)^. Additionally, a reproducible 150-probe-set RNA classifier has been established for multi-tissue aging in humans, including skeletal muscles^10)^.

Despite these advances, the existing tools often require large gene sets, which limits their clinical translation because of the high cost and complexity involved. In this study, we developed a gene panel to detect senescence-associated cells in a simple and highly accurate manner to overcome the drawbacks of current approaches.

## Materials and Methods

### Data acquisition

Publicly available single-cell and single-nucleus RNA sequencing (scRNA-seq and snRNA-seq) datasets from male and female C57BL/6J mouse skeletal muscles were obtained from the DRYAD Repository^11)^. These data were originally generated over 18 previous studies, resulting in a compendium of over 365,000 cells and nuclei across multiple age groups and injury conditions, which were assigned to 23 clusters. Because batch correction across these studies was performed by the original authors of these studies as part of dataset construction, no additional batch correction was applied in the present study. This compendium was generated using a unified processing and integration framework, ensuring consistency across studies. The muscle sources used in this study were the tibialis anterior (TA) and hindlimb muscles. This dataset was used for candidate gene identification and model training. A second, fully independent dataset was used for validation and comparison with an existing multigene marker panel. The validation dataset was deposited separately in DRYAD and shares no samples or libraries with the training dataset. As the two datasets were not integrated, no cross-dataset batch correction was applied. All downstream analyses were performed using R (v4.5.0) and Seurat packages (v5.3.0).

### Data preprocessing, clustering, and candidate gene filtering

The pre-assigned clusters were subsequently consolidated into 15 biologically related groups by merging similar cell types. Specifically, Monocyte (Patrolling), Monocyte (Inflammatory), and Monocyte (Cxcl10^+^) were merged as monocytes; Endothelial (Capillary), Endothelial (Vein), and Endothelial (Artery) as endothelial cells; Fibro-adipogenic progenitors (FAPs; pre-remodeling), FAPs (stem), and FAPs (adipogenic) as FAPs; M2 macro (Cx3cr1^lo^) and M2 macro (Cx3cr1^hi^) as macrophages; and Myonuclei (type 2b) and Myonuclei (type 2x) as myonuclei.

For the downstream analysis, uninjured skeletal muscle cells were extracted from male C57BL/6J mice aged 2 and 24 months. Differentially expressed genes (DEGs) between the 2- and 24-month-old mice within each of the 15 clusters were identified using the FindMarkers function in Seurat with the Wilcoxon rank-sum test. The minimum log2 fold-change threshold was 0.25. Genes with a Bonferroni-adjusted P-value < 0.05 were retained as significant DEGs within each cluster. Aging-associated genes were selected by retaining only genes that were considered DEGs in at least nine clusters. This threshold was chosen to obtain a manageable number of broadly expressed candidate genes suitable for downstream machine learning. This strategy prioritizes genes that are reproducibly associated with age across heterogeneous cellular contexts over those differentially expressed in a single cell type and thereby enables the construction of a classifier applicable at the tissue level. We reasoned that such cross-cluster-reproducible genes are less likely to reflect cell-type-specific artifacts or context-dependent responses and are, therefore, more suitable candidates for a compact and generalizable classifier. Genes whose names began with *Mt*, *Rp*, or *Gm* were excluded to remove mitochondrial, ribosomal, and pseudogene artifacts.

### Machine learning and model construction

Machine learning and model training were performed using R with Caret (v7.0) and pROC (v1.18.5). Sample metadata and count matrices were randomly split into training (70%) and test (30%) sets. The features for model training were selected from the DEGs identified in the previous step. Recursive feature elimination was applied to all classification models to identify the optimal feature subset.

The model performance was evaluated using the area under the receiver operating characteristic curve (ROC-AUC). The classification algorithms included logistic regression with RIDGE and LASSO regularization, support vector machines with linear and radial basis function kernels (SVMLin and SVMRAD, respectively), and random forest (RF). Genes were added iteratively from the top-ranked features until the ROC-AUC exceeded 0.9, at which point the algorithm was halted. This threshold was adopted based on the widely used classification scheme presented in a review wherein an AUC of 0.9-1.0 was considered “outstanding” discrimination^12)^. In addition, this cutoff was selected to balance performance and model simplicity, as further increases in AUC required additional genes with only marginal improvement in classification performance.

### Comparison with previous multigene panel

The previous model was originally developed as a continuous senescence score rather than as an age classifier^9)^. The precomputed senescence scores provided with the validation dataset were used directly without recalculation. To enable direct comparison with our four-gene model under a unified binary classification framework, the continuous scores were binarized using a threshold determined based on the Youden index on the ROC curve. The metrics reported here should, therefore, be interpreted within this binary classification context, and they may not fully reflect the intended performance of the original panel as a continuous senescence indicator.

## Results

### Dataset overview and aging-associated candidate gene identification

**Figure 1:**
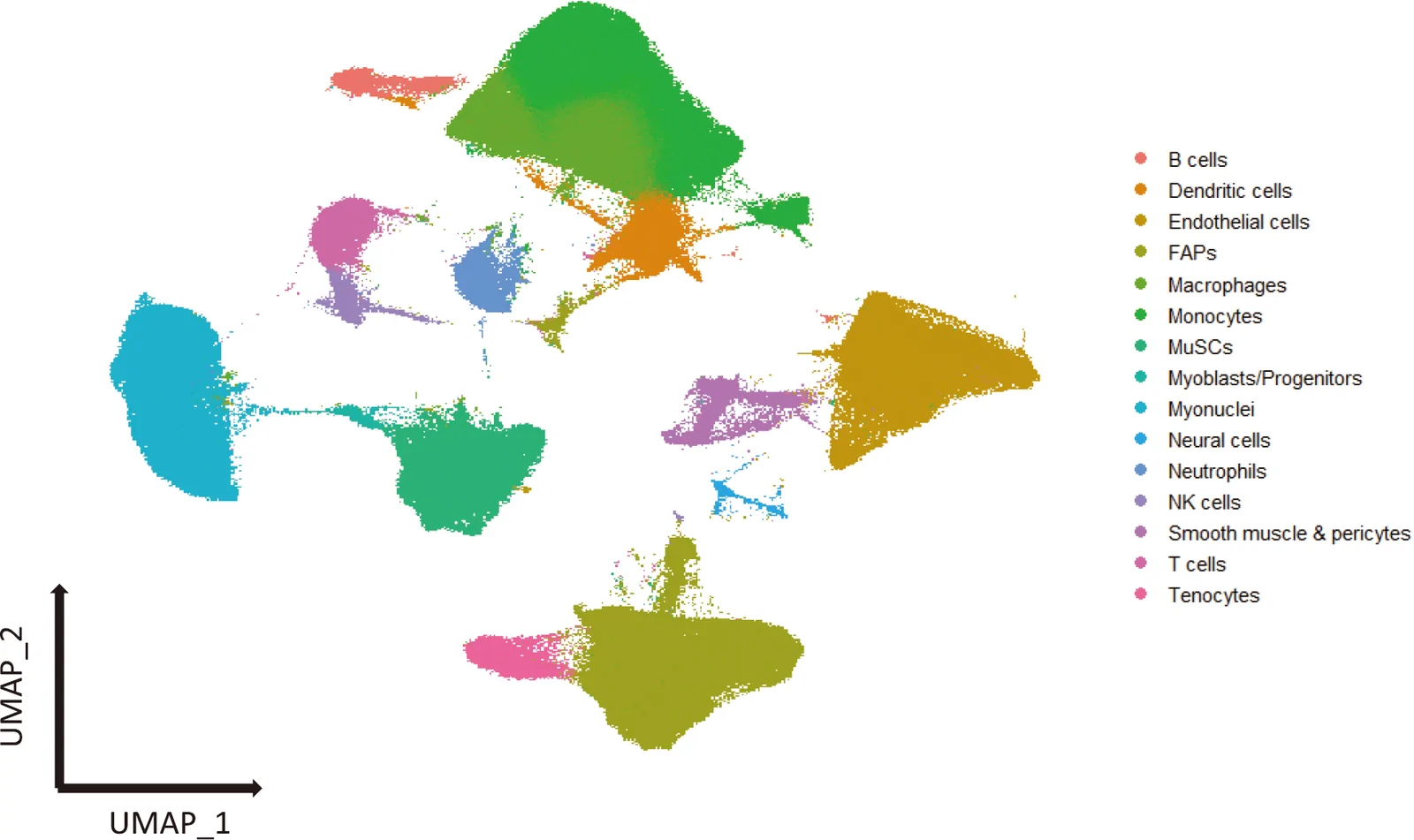
Uniform Manifold Approximation and Projection (UMAP) visualization of cells from mouse skeletal muscle. Cells are colored by cell type, consolidated into 15 cell populations.

An open-access dataset comprising 365,011 cells initially organized into 23 clusters was utilized and visualized using Uniform Manifold Approximation and Projection (UMAP) (Figure 1). Subsequently, these clusters were consolidated into 15 biologically related groups. The data of uninjured skeletal muscle cells from male mice aged 2 and 24 months were extracted for further analysis.

DEGs within each cluster were identified by comparing cells from 2- and 24-month-old mice. DEGs in at least nine clusters were selected, yielding 52 genes. After excluding genes with *Mt-*, *Rp-*, and *Gm-* prefixed to their names, 30 candidate genes remained for downstream analysis, *Aes, Anxa1, Anxa11, Atf3, Atp5l, Casp4, Eef1a1, Erdr1, Fosb, Grina, Hmox1, Ifitm2, Ifitm3, Irf1, Jund, Lmna, Maff, Malat1, Mir1931, Rhob, Rnd1, Sepw1, Spag7, Tagln2, Tmem176a, Tmem176b, Tpt1, Vim, Wdr89, and Zfp36*.

### Construction and validation of a four-gene signature marking senescence-associated cells in skeletal muscle

The training and testing datasets were analyzed using machine learning models. Five machine learning models were evaluated to determine the minimum number of genes required to achieve an ROC-AUC exceeding 0.9. Both LASSO and RIDGE required six genes each; SVMRad required five genes; and SVMLin and RF each achieved a threshold with only four genes. SVMLin was selected as the optimal model based on its superior performance compared with RF (AUC = 0.903 vs 0.902, respectively) (Table 1). This resulted in a four-gene signature comprising *Wdr89, Malat1, Jund*, and *Zfp36*. This signature was subsequently applied to skeletal muscle cells from mice of different ages (3, 4, 7, 18, 20, and 21 months). The proportion of cells classified as “Old” progressively increased with age: 44.7% at 3 months, 79.1% at 7 months, and 94.6% at 18 months (Table 2).

**Table 1:** Comparison of machine learning models

	Gene list	ROC-AUC
Logistic regression (RIDGE)	*Malat1, Wdr89, Erdr1, Mir1931, Spag7, Anxa11*	0.906
Logistic regression (LASSO)	*Malat1, Wdr89, Erdr1, Mir1931, Spag7, Anxa11*	0.906
SVMLin	*Malat1, Wdr89, Jund, Zfp36*	0.903
SVMRAD	*Malat1, Wdr89, Jund, Zfp36, Ifitm2*	0.902
Random forest	*Malat1, Wdr89, Jund, Tpt1*	0.902

The minimum number of genes required to achieve ROC-AUC > 0.9 and the corresponding AUC values are shown for each line.

**Table 2:** Age-dependent classification using the four-gene model

Age (months)	Old	Young	Total	Percentage
2	3901	10230	14131	27.6
3	1993	2461	4454	44.7
4	3820	919	4739	80.6
7	4758	1259	6017	79.1
18	8489	487	8976	94.6
20	10343	1343	11686	88.5
21	4159	752	4911	84.7
24	8449	1714	10163	83.1

The percentage of cells classified as Old is shown for each age group.

### Cell-type performance of the four-gene signature

Across major cell types, the proportion of cells classified as “Old” increased with age, although baseline levels varied substantially between cell types (Supplementary Table 1). For example, MuSCs and immune cell populations showed a more gradual increase across age groups, whereas myonuclei exhibited a high proportion of cells classified as “Old” even in younger samples. Despite these differences in baseline levels, an age-associated upward trend was observed consistently across nearly all cell types.

To further assess whether this pattern was independent of cellular composition, we fitted a pooled quasibinomial logistic regression model including cell type as a covariate. Age remained a highly significant predictor of “Old” classification across all groups (Supplementary Table 2), indicating that the association between the four-gene signature and aging is largely preserved after accounting for differences in cell-type composition. Although the sample sizes for some cell types were limited, and the results should therefore be interpreted with caution, the consistent trends observed across major cell populations support the robustness of these findings.

### Correlation between the four-gene signature and canonical senescence markers across ages

The correlation between the four-gene signature and established senescence markers (*Cdkn1a, Cdkn2a, Ccl2*, and *Il6*) was examined by comparing their expression levels in cells as “Young” versus “Old” within each age group. *Cdkn1a* expression was significantly higher in cells classified as “Old” across all age groups. For *Il6*, cells classified as “Young” only showed higher expression at 2 months, whereas cells classified as “Old” exhibited significantly higher expression at all other ages (Table 3).

**Table 3:** Age-stratified differential expression in cells classified as “Young” or “Old”

Gene	Age (months)	log2FC	P-value	Adj P-value	Pct (Old)	Pct (Young)
*Cdkn1a*	2	1.11	3.49 × 10^-105	1.12 × 10^-103	0.641	0.44
	3	0.993	1.12 × 10^-37	8.99 × 10^-37	0.686	0.549
	4	1.54	1.63 × 10^-59	2.61 × 10^-58	0.77	0.472
	7	0.805	6.34 × 10^-23	2.90 × 10^-22	0.623	0.482
	18	0.399	8.57 × 10^-4	0.00159	0.738	0.608
	20	0.668	7.55 × 10^-17	2.42 × 10^-16	0.462	0.341
	21	0.762	1.24 × 10^-17	4.41 × 10^-17	0.83	0.691
	24	0.987	1.61 × 10^-50	1.71 × 10^-49	0.772	0.583
*Cdkn2a*	2	-0.576	0.153	0.196	0.00128	0.00254
	3	-0.191	0.669	0.738	0.00401	0.00488
	4	-1.24	0.0541	0.0722	0.00314	0.00762
	7	20	0.304	0.374	0.000841	0
	18	0.902	0.502	0.574	0.00401	0.00205
	20	-0.509	0.809	0.835	0.00474	0.00521
	21	-0.213	0.923	0.923	0.00505	0.00532
	24	-1.31	0.0204	0.0297	0.00604	0.0111
*Ccl2*	2	-1.36	2.29 × 10^-33	1.47 × 10^-32	0.0874	0.165
	3	-0.441	0.00271	0.00434	0.202	0.236
	4	0.962	3.31 × 10^-9	9.63 × 10^-9	0.26	0.169
	7	0.482	0.035	0.0487	0.0509	0.0365
	18	0.423	9.77 × 10^-4	0.00165	0.331	0.251
	20	0.832	8.95 × 10^-4	0.00159	0.0485	0.0283
	21	-0.0275	0.734	0.783	0.376	0.362
	24	0.101	0.319	0.378	0.299	0.286
*Il6*	2	-0.408	6.86 × 10^-7	1.57 × 10^-6	0.112	0.144
	3	0.222	0.00381	0.0058	0.249	0.209
	4	1.28	1.89 × 10^-21	7.56 × 10^-21	0.298	0.137
	7	1.3	1.26 × 10^-5	2.69 × 10^-5	0.0441	0.0175
	18	0.788	1.43 × 10^-8	3.81 × 10^-8	0.373	0.23
	20	1.54	1.90 × 10^-4	3.80 × 10^-4	0.0253	0.00894
	21	0.617	2.62 × 10^-8	6.44 × 10^-8	0.436	0.319
	24	1.02	1.99 × 10^-32	1.06 × 10^-31	0.413	0.256

For each gene and age, log2FC (log2 fold change; Old vs. Young) and corresponding *P*-value are shown, together with the adjusted *P*-value (Adj *P*-value). Pct (Old) and Pct (Young) indicate the proportion of cells with detectable expression (expression > 0) in the “Young” and “Old” group, respectively. Positive log2FC indicates higher expression in “Old” relative to “Young.”

### Comparison of the four-gene signature with an established multi-gene panel

**Figure 2:**
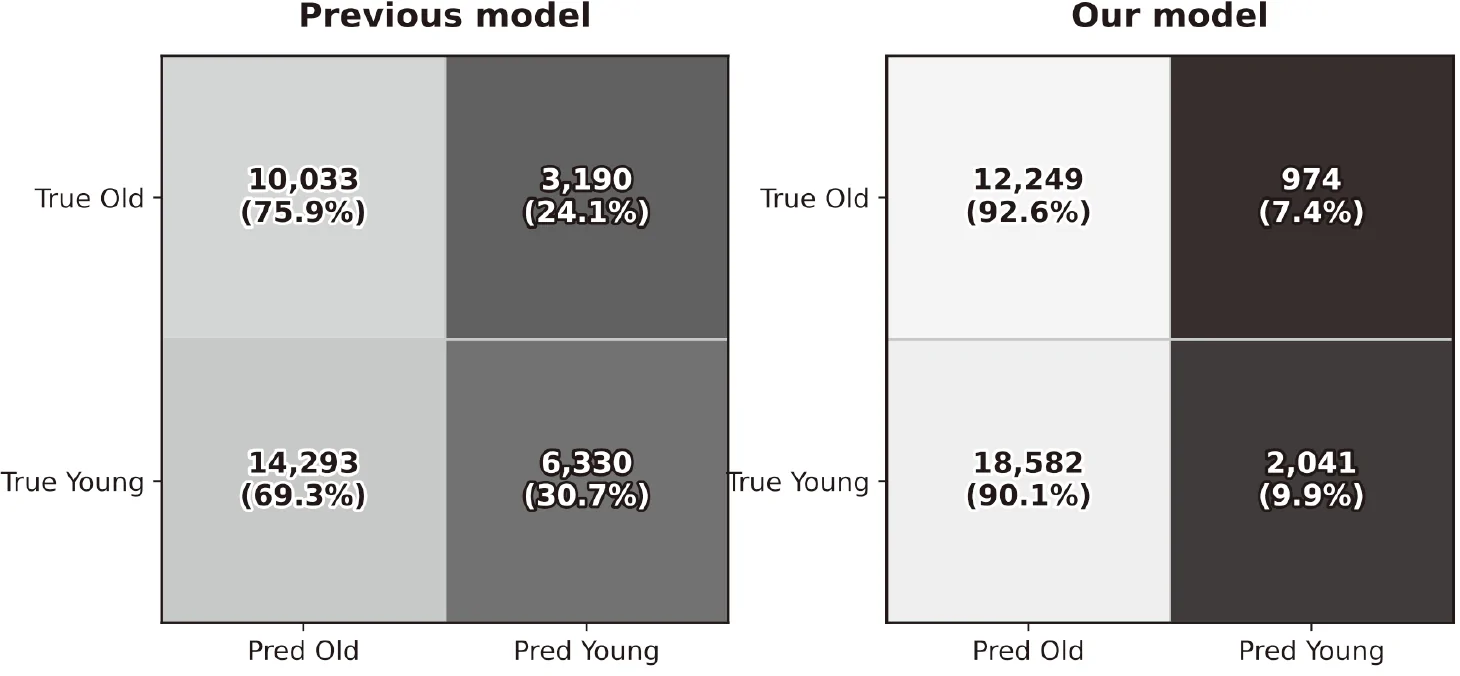
Performance comparison between the four-gene model and the previous model. Cell counts are shown in each table.

The performance of the four-gene model was compared with that of an existing multigene marker panel across multiple classification metrics (Figure 2)^9)^. The four-gene model achieved higher sensitivity than the previous panel (0.926 vs. 0.759), but exhibited lower specificity (0.099 vs. 0.307). The lower specificity indicates a higher false-positive rate, which represents a limitation of the model when used by itself. Precision was numerically similar between the two models (0.397 vs. 0.412), and the F1 score of the four-gene model (0.556) was slightly higher than that of the previous panel (0.534). The overall accuracy was lower for the four-gene model (0.422 vs. 0.483), which may reflect its higher false-positive rate.

## Discussion

In the present study, a combination of scRNA-seq data and machine learning was used to develop a four-gene signature (*Wdr89, Malat1, Zfp36*, and *Jund*) for identifying senescence-associated cells in skeletal muscle tissues. Although the specificity (9.9%) was lower than that of the previous multigene panel (30.7%), the model showed high sensitivity (92.6%). Despite this profile, the two models achieved comparable precision (0.397 vs. 0.412) and F1 score (0.556 vs. 0.534), indicating that the four-gene model is positioned not as a definitive classifier of individual senescent cells but as a rapid, cost-effective screening tool for senescence-associated states. This performance profile—high sensitivity with reduced specificity—may be appropriate for screening-oriented uses, where capturing a broad set of candidate senescent cells is prioritized over minimizing false positives, provided that downstream validation or filtering steps are applied to mitigate the high false-positive rate.

The four genes were selected based on their differential expression across age groups and evaluated using multiple machine learning algorithms. *Malat1*, a long non-coding RNA, regulates *Sirt1* expression and contributes to the prevention of muscle atrophy^13)^. *Sirt1* plays key roles in protein homeostasis, apoptosis regulation, and mitochondrial function, and is implicated in the prevention of sarcopenia^14)^. *Jund*, a component of the AP-1 transcription factor complex, is implicated in stress response and cellular senescence^15, 16)^. In skeletal muscle, JunD-containing AP-1 complex regulates satellite cell expansion^17)^. Its dysregulation is, therefore, expected to disrupt the reversible quiescence- activation cycle, impairing the ability of satellite cells to maintain quiescence and avoid senescence. Furthermore, the AP-1 complex has been implicated in the regulation of the senescence-associated secretory phenotype (SASP)^18)^. Age-dependent changes in *Jund* expression have also been reported in other tissues, which is indicative of its broader role in age-related process^19)^. *Zfp36*, which encodes an RNA-binding protein that destabilizes mRNA targets, is upregulated in certain senescent or inflammatory contexts, and modulates inflammatory responses^20)^. In skeletal muscle, *Zfp36* contributes to the maintenance of satellite cell quiescence by promoting the decay of *MyoD* mRNA, thereby regulating the timing of satellite cell activation and muscle regeneration^21)^. As impaired regulation of satellite cells is a hallmark of aged skeletal muscle, age-related alteration in *Zfp36* expression may contribute to, or reflect, the decline in satellite cell function observed during skeletal muscle senescence. *Wdr89*, which encodes a WD repeat-containing protein, has been reported to be associated with cancer-related process, although its functional role remains poorly characterized and it has not been directly linked with senescence^22)^. The identification of Wdr89 through a purely data-driven approach highlights the potential of machine learning to uncover novel candidates that would not have been prioritized based on prior knowledge alone. Collectively, the identification of these genes suggests convergence on pathways related to stress response, inflammatory regulation, and satellite cell maintenance, all of which are closely associated with the establishment and progression of senescence-associated cell states in skeletal muscle.

Skeletal muscle is predominantly composed of multinucleated muscle fibers that are not captured as individual cells^23)^. This structural feature makes snRNA-seq preferable for studying transcriptional changes within fibers^24)^. However, constructing a model using snRNA-seq requires more than 100 genes to achieve a comparable classification accuracy. In contrast, the scRNA-seq approach in this study enabled the identification of a small set of discriminative genes, reflecting the substantial contribution of surrounding non-myofiber cells, such as immune cells and FAPs, to overall skeletal muscle aging rather than the intrinsic aging of muscle fibers. These observations highlight the importance of the cellular microenvironment in skeletal muscle senescence.

The present study had some limitations. The analyzed datasets were derived from the TA and hindlimb muscles; however, individual muscles within the hindlimb were not consistently distinguished. As a result, although these datasets likely include data from muscle types with differing fiber- type composition, the present analysis does not explicitly resolve slow- versus fast-twitch muscle identity. Consequently, the extent to which the identified gene signature reflects fiber-type-specific aging processes remains unclear. In addition, the findings of this study are limited to mouse skeletal muscle and experimental validation of the functional roles of the four selected genes remains lacking. Further studies are required to evaluate the applicability of this model across different muscles, tissues, and species and to confirm the biological relevance of these candidate genes in vivo.

Nonetheless, this four-gene model is a promising, rapid screening tool for skeletal muscle aging, balancing sensitivity and economy, while opening new approaches for investigating muscle senescence.

## Data availability

Publicly available single-cell RNA-seq datasets were utilized in this study. The primary dataset used for the identification of 30 aging-associated candidate genes and the construction of the four-gene signature through machine learning approaches is deposited in DRYAD under accession https://doi.org/10.5061/dryad.t4b8gtj34. The dataset used for validation and comparison with the existing multigene marker panel is deposited in DRYAD under accession https://doi.org/10.5061/dryad.kkwh70sbv. Materials and protocols are available from the corresponding author upon reasonable request.

## Author contributions

TK conceptualized the study, conducted the experiments, and wrote the original manuscript. YY provided supervision throughout the project and contributed to the original draft preparation. EAH supervised the overall work and revised the manuscript. All authors read and approved the final version of the manuscript.

## Conflicts of interest statement

The authors declare that there are no conflicts of interest. All figures presented in this manuscript are original and have not been reproduced or adapted from previously published sources. Eri Arikawa-Hirasawa, one of the advisory board members of JMJ, was not involved in the peer review or decision-making process for this paper.

## Supplementary Material

**Supplemental Table 1:** Proportion of cells classified as “Old” based on the four-gene signature, stratified by cell types and age

Cell type	2mo	3mo	4mo	7mo	18mo	20mo	21mo	24mo
MuSCs	10.2% (n = 1019)	14.7% (n = 217)	67.5% (n = 348)	72.8% (n = 103)	87.2% (n = 500)	87.5% (n = 160)	78.3% (n = 244)	71.0% (n = 403)
Myoblasts/Progenitors	25.0% (n = 8)	50.0% (n = 10)	75.0% (n = 4)	100.0% (n = 1)	100.0% (n = 8)	75.0% (n = 4)	100.0% (n = 1)	100.0% (n = 8)
Myonuclei	85.4% (n = 82)	100.0% (n = 88)	100.0% (n = 25)	99.0% (n = 514)	95.2% (n = 62)	99.5% (n = 1281)	100.0% (n = 57)	100.0% (n = 103)
FAPs	22.6% (n = 2135)	40.5% (n = 1312)	92.3% (n = 1150)	74.9% (n = 1819)	96.5% (n = 2620)	84.7% (n = 2412)	90.6% (n = 1675)	90.5% (n = 2790)
Tenocytes	19.9% (n = 873)	41.9% (n = 341)	90.2% (n = 367)	74.7% (n = 154)	94.6% (n = 836)	76.9% (n = 316)	76.7% (n = 425)	81.7% (n = 699)
Endothelial cells	53.8% (n = 4412)	66.7% (n = 974)	87.5% (n = 1538)	82.3% (n = 2640)	96.3% (n = 3276)	89.9% (n = 6229)	86.3% (n = 1858)	85.7% (n = 3848)
Smooth muscle & pericytes	18.9% (n = 593)	26.5% (n = 155)	71.5% (n = 193)	65.1% (n = 415)	87.3% (n = 543)	85.8% (n = 570)	62.1% (n = 169)	65.3% (n = 473)
Macrophages	16.5% (n = 345)	33.3% (n = 93)	76.9% (n = 91)	83.0% (n = 47)	93.9% (n = 313)	94.0% (n = 84)	80.7% (n = 150)	79.8% (n = 228)
Monocytes	22.7% (n = 88)	87.5% (n = 16)	89.6% (n = 48)	87.0% (n = 23)	97.9% (n = 383)	91.8% (n = 98)	88.5% (n = 87)	88.9% (n = 207)
Dendritic cells	14.5% (n = 777)	43.0% (n = 179)	74.9% (n = 171)	95.5% (n = 22)	93.4% (n = 61)	100.0% (n = 23)	78.7% (n = 47)	79.8% (n = 228)
Neutrophils	30.6% (n = 36)	46.7% (n = 15)	60.0% (n = 10)	100.0% (n = 6)	98.8% (n = 83)	100.0% (n = 27)	81.8% (n = 11)	84.4% (n = 64)
B cells	10.1% (n = 2018)	41.8% (n = 569)	58.7% (n = 443)	72.7% (n = 22)	90.9% (n = 33)	83.7% (n = 49)	66.3% (n = 95)	69.8% (n = 577)
T cells	7.5% (n = 1279)	26.3% (n = 350)	48.1% (n = 270)	72.0% (n = 25)	91.3% (n = 46)	84.6% (n = 39)	59.0% (n = 39)	63.5% (n = 375)
NK cells	11.9% (n = 243)	47.1% (n = 87)	53.1% (n = 49)	75.0% (n = 4)	78.6% (n = 14)	80.0% (n = 10)	30.0% (n = 10)	71.1% (n = 76)
Neural cells	22.9% (n = 223)	6.2% (n = 48)	53.1% (n = 32)	58.6% (n = 222)	74.7% (n = 198)	65.6% (n = 384)	55.8% (n = 43)	60.7% (n = 84)

Each cell shows the percentage of cells classified as “Old” and the total number of cells analyzed (n).

**Supplemental Table 2:** Age-group-specific odds ratios for “Old” classification from the pooled quasibinomial logistic regression model

Age group (months)	OR (vs. 2 months)	95% CI	*P*-value
3	2.22	1.69-2.91	1.18e-07
4	12.18	8.92-16.65	1.75e-28
7	7.12	5.36-9.46	3.09e-24
18	42.09	28.96-61.18	1.12e-35
20	13.51	10.37-17.59	3.73e-35
21	12.47	8.98-17.31	2.96e-27
24	12.41	9.71-15.86	1.44e-36

Odds ratios (OR) are shown for each age group relative to the 2-month group, which served as the reference category. The pooled model included cell type as a covariate to account for differences in cellular composition across samples.
